# Estimated projection of oral cavity and oropharyngeal cancer deaths in Spain to 2044

**DOI:** 10.1186/s12903-022-02487-6

**Published:** 2022-10-14

**Authors:** Pedro Infante-Cossio, Antonio-Jose Duran-Romero, Antonio Castaño-Seiquer, Rafael Martinez-De-Fuentes, Jose-Juan Pereyra-Rodriguez

**Affiliations:** 1 Department of Oral and Maxillofacial Surgery, Virgen del Rocio University Hospital, University of Seville, Seville, Spain; 2 Department of Dermatology, Virgen del Rocio University Hospital, University of Seville, Seville, Spain; 3 Department of Preventive and Community Dentistry, School of Dentistry, University of Seville, Seville, Spain; 4 Department of Prosthodontics, School of Dentistry, University of Seville, Seville, Spain

**Keywords:** Projection, Mortality, Oral cavity cancer, Oropharynx cancer, Spain

## Abstract

**Background:**

Oral cavity cancer (OCC) and oropharyngeal cancer (OPC) are two common malignancies whose mortality is worryingly increasing worldwide. However, few studies have estimated the mortality trends for these cancers in the coming years. This study analysed the mortality rates for OCC and OPC observed between 1980 and 2019 to generate a predictive model for the next 25 years in Spain.

**Methods:**

Mid-year population data and death certificates for the period 1980–2019 were obtained from the Spanish National Institute of Statistics. The Nordpred program (Norwegian Cancer Registry, Oslo, Norway) was used to calculate adjusted mortality rates as well as estimated mortality projections with an age-period-cohort model for the period 2020–2044.

**Results:**

The specific mortality rate per 100,000 inhabitants for OCC decreased from 2.36 (1980–1984) to 2.17 (2015–2019) and is expected to decline to 1.68 (2040–2044), particularly in males. For OPC, mortality rates rose from 0.67 (1980–1984) to 1.23 (2015–2019) and are projected to drop to 0.71 (2040–2044). In the group of females > 65 years predictions showed rising mortality rates for both OCC and OPC. The predictive model projects more deaths in females than in males for OCC in the period 2040–2044, while deaths for OPC will decrease in males and gradually increase in females.

**Conclusions:**

Although OCC mortality rates have been found to decrease in males in the last observed decades, there is still room to improve them in females > 65 years in the future by promoting campaigns against smoking and alcohol consumption. OPC mortality will become a growing health problem. Vaccination campaigns for the prevention of human papillomavirus-associated cancers may have a long-term impact on the mortality of these cancers, which should be evaluated in upcoming studies.

**Clinical relevance:**

Our findings highlighted the importance of closely monitoring OCC and OPC mortality rates in the coming years by age group and sex, and the need to continue preventive measures against the main known risk factors, such as tobacco, alcohol, and human papillomavirus infection.

**Supplementary Information:**

The online version contains supplementary material available at 10.1186/s12903-022-02487-6.

## Background

Oral cavity cancer (OCC) and oropharyngeal cancer (OPC) are two malignant tumours closely related through the narrow topographical boundary between the mouth and the oropharynx. Squamous cell carcinoma arising from the superficial epithelium of the oral cavity and oropharynx accounts for more than 90% of all cancers in this region. Recent evidence supports that these cancers could be considered two different diseases in terms of etiopathogenesis, treatment, and prognosis [1]. Although they classically share common non-genetic risk factors, such as tobacco and alcohol consumption, human papillomavirus (HPV) is currently recognized as a specific risk factor primarily for OPC.

OCC and OPC represent a growing health concern since, in 2018, they were estimated to cause 177,400 deaths (119,700 males, 7,700 females) and 51,000 deaths (42,100 males, 8,900 females) worldwide, respectively [2]. Considering all locations together, neoplasms of the oral cavity, oropharynx, and pharynx are the ninth leading cause of cancer death globally [3]. Available data on mortality rates due to OCC and OPC in Spain are scarce. It has been reported that the mortality rates of these two related cancers reached their highest values in the 1990s, when they began to fall and have stabilized to the present [4]. However, in the last two decades, while OCC mortality rates have decreased markedly in males, a slight increase has been observed in females. On the contrary, a steady increase in OPC mortality has been observed in both sexes over the last decade.

Knowledge of OCC and OPC mortality rates and trends in large populations is a powerful tool for the planning process of public health strategies and for defining future research priorities. The government mortality registry available in Spain provides a high quality data source that allows accurate mortality analysis. This reliable data can be used to make large-scale predictions, showing trends not observable in small studies or clinical trials. Very few studies have reported predictions of OCC and OPC mortality for the next several years, and to our knowledge, no study has ever been conducted comparing the estimated mortality projections for these two related cancers separately and in combination. In Spain there are no specific reports that predict the future trends of mortality due to OCC and OPC. Consequently, this study aimed to analyse the mortality rates in the last 40 years to generate a predictive model of OCC and OPC mortality in Spain up to 2044.

## Methods

### Data collection

Mid-year population data and death certificates for the period 1980–2019 were obtained from the Spanish National Institute of Statistics (INE) (http://www.ine.es). Population data for the period 2020–2044 were also obtained from the official population projection figures provided by the INE. For the present study, death certificates obtained from microdata files which included OCC or OPC as the cause of the dead, were included in the study. On death certificates, the cause of the death was coded according to the 9th or 10th edition of the International Classification of Diseases (ICD-9, ICD-10). The codes included for OCC were the following: lip (140, C00), tongue (141, C02 excluding C02.4), gingiva (143, C03), floor of the mouth (144, C04), and other sites of the mouth (145, C05-06 excluding C05.1); and for OPC: base of the tongue (C01), lingual tonsil (C02.4), soft palate (C05.1), tonsil and oropharynx (146, C09-10). Malignant neoplasms of the nasopharynx, hypopharynx, and major salivary glands were excluded.

### Statistical analysis

The Nordpred package (Norwegian Cancer Registry, Oslo, Norway) [5] with the statistical program R [6] was used to calculate standardized and age-specific mortality rates, as well as the projection of mortality. For standardization, the direct method and the European standard population of 2013 were used. The Nordpred R package was employed to calculate an age-period-cohort model. Data were collected in 5-year blocks. The most recent 10-year linear trend was extrapolated, attenuating the slope by 25% and 50% in the second and third prediction periods, and by 75% in the fourth and fifth prediction periods, as recommended by the authors after empirical validation [5]. The prediction results were presented for total observed and projected deaths for each period. Adjusted mortality rates were calculated from age-specific rates for each period. These specific mortality rates were calculated by dividing the projected number of deaths by the mid-term population for each age range, multiplying by 100,000. The mortality rate between sexes was calculated and stratified by age groups. Annual changes in the number of deaths were calculated for the latest projected period (2040–2044) compared to the lasted observed period (2015–2019), where the proportion of this change could be due to changes in the risk of death for OCC and OPC or due to demographic changes (size or structure of the population). These two components can be non-zero and have a positive or negative direction. Based on our data, there was no population aging during the study period, so we attribute our results to real changes in mortality trends. Nordpred used the Power5 and Poisson age-period-cohort models to calculate the prediction of mortality between 2020 and 2044 [7]. Since statistical methods based on age-period-cohort models often overestimated the number of cases due to their exponential growth over time, we decided to use the Power5 function to level this out and improve our main predictions.

## Results

### Observed mortality trends

**Fig. 1:**
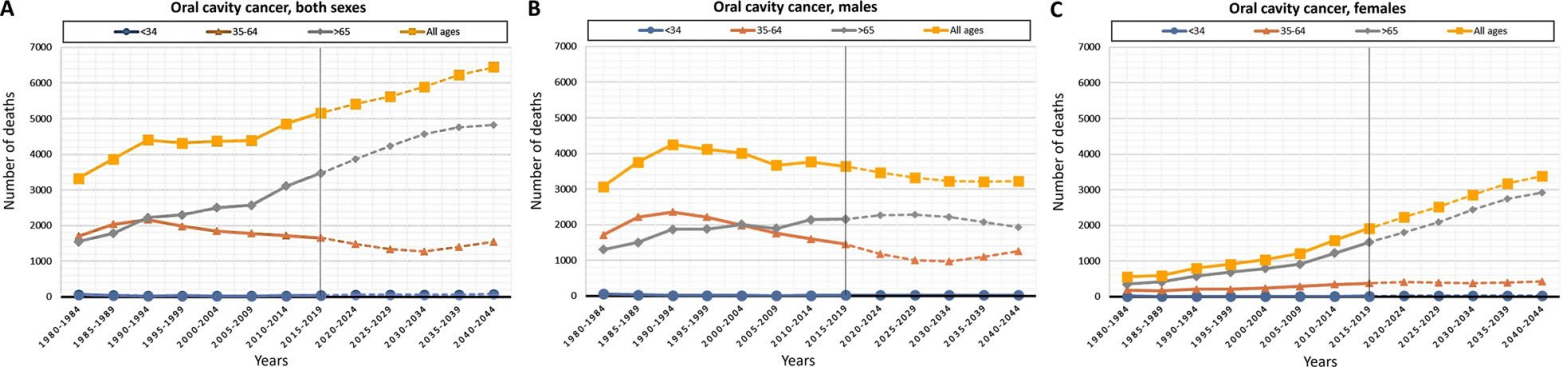
Graphical representation of the number of deaths observed (period 1980–2019) (solid line) and projected (period 2020–2044) (dash line) by age groups (< 34, 35–64 and > 65 years) for OCC: **A** both sexes; **B** males; **C** females

**Fig. 2:**
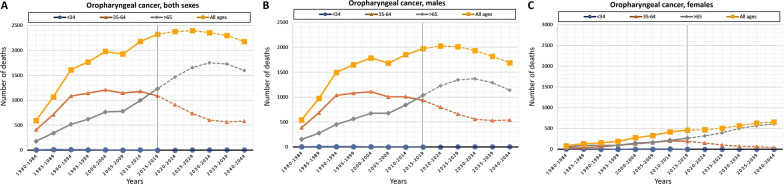
Graphical representation of the number of deaths observed (period 1980–2019) (solid line) and projected (period 2020–2044) (dash line) by age groups (< 34, 35–64 and > 65 years) for OPC: **A** both sexes; **B** males; **C** females

Table 1 shows observed deaths in the period 1980–2019 according to sex and sub-site. In total, 34,708 OCC deaths (supplementary Table 1, Additional file 1) and 18,216 OPC deaths (supplementary Table 2, Additional file 1) were recorded. The number of deaths for OCC and OPC rose from 3,324 and 935 (period 1980–1984) to 5,163 and 2,828 (period 2015–2019), respectively. According to sex, there were 26,069 deaths in males and 8,639 in females for OCC (male/female ratio: 3/1), and 16,170 deaths in males and 2,046 in females for OPC (male/female ratio: 8/1). In Figs. 1 and 2, these data are represented graphically by categorizing the number of deaths by sex and age groups (< 34, 35–64 and > 65 years).

**Table 1:** Observed deaths (period 1980–2019) by sex and sub-site

	Both sexes		Males		Females	
	n	Mean (SD)	n	Mean (SD)	n	Mean (SD)
*OCC*						
Lip	2872	75.61 (12.23)	2357	73.84 (12.05)	515	83.68 (9.46)
Tongue*	15,764	66.08 (13.47)	11,745	63.93 (12.39)	4019	72.38 (14.95)
Gingiva	1257	69.06 (15.89)	704	65.87 (15.57)	553	73.12 (15.37)
Floor of mouth	5921	63.31 (12.12)	5132	62.18 (11.32)	789	70.71 (14.37)
Other sites of the mouth**	8894	67.59 (13.98)	6613	64.65 (12.91)	2857	74.41 (13.98)
Total	34,708	66.90 (13.72)	26,551	64.70 (12.75)	8733	73.60 (14.39)
*OPC*						
Base of the tongue	4262	62.97 (11.91)	3783	62.20 (11.29)	479	69.10 (14.64)
Soft palate	449	65.42 (13.21)	376	64.08 (12.53)	73	72.33 (15.70)
Lingual tonsil	62	63.03 (11.74)	54	62.67 (11.50)	8	65.50 (13.86)
Tonsil	5305	61.69 (11.98)	4729	61.20 (11.43)	576	65.68 (15.22)
Oropharynx	8138	62.50 (11.36)	7228	62.05 (10.92)	910	66.10 (13.89)
Total	18,216	62.45 (11.74)	16,170	61.88 (11.21)	2046	66.90 (14.60)

*Excluding lingual tonsil

**Excluding soft palate

**Fig. 3:**
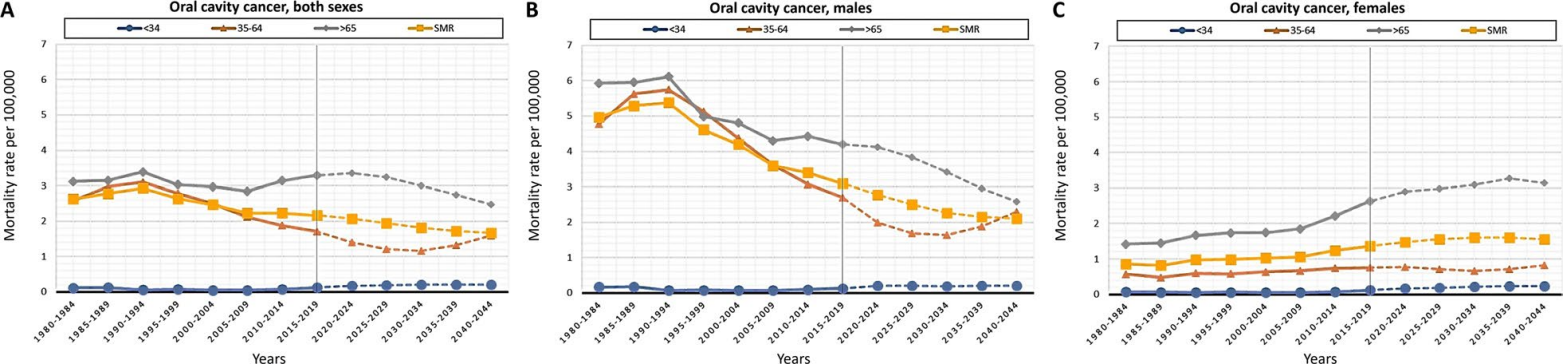
Graphical representation of the observed (period 1980–2019) (solid line) and projected (period 2020–2044) (dash line) age-specific mortality rates per 100,000 inhabitants (European population 2013) by age groups (< 34, 35–64 and > 65 years) for OCC: **A** both sexes; **B** males; **C** females

**Fig. 4:**
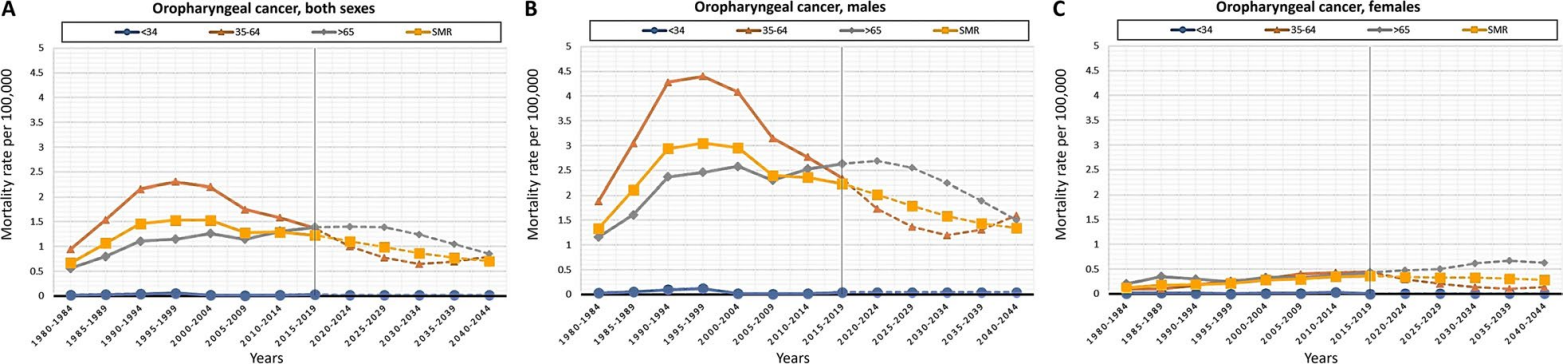
Graphical representation of the observed (period 1980–2019) (solid line) and projected (period 2020–2044) (dash line) age-specific mortality rates per 100,000 inhabitants (European population 2013) by age groups (< 34, 35–64 and > 65 years) for OPC: **A** both sexes; **B** males; **C** females

Supplementary Tables 3 and 4 (Additional file 1) show the estimated projection of the specific mortality rates per 100,000 inhabitants, according to 5-year blocks and sex. The OCC mortality rate decreased from 2.63 (period 1980–1984) to 2.17 (period 2015–2019). In males, the mortality rate decreased from 4.97 to 3.09, while in females increased from 0.86 to 1.36. The OPC mortality rate rose from 0.67 deaths (period 1980–1984) to 1.23 deaths (period 2015–2019). The sex-specific mortality rate grew up in both sexes, from 1.33 to 2.23 deaths in males and from 0.12 to 0.36 in females. For OCC, mortality rates showed a slight decline and a subsequent levelling off throughout the period 1980–2019 in all age groups (Fig. 3A). This drop was more pronounced in males > 65 years (Fig. 3B). In contrast, an increase in mortality rates was detected in females > 65 years from the period 2005–2009 (Fig. 3C). Regarding OPC mortality rates, a constant increase was noted from the beginning of the observed period, especially in 35–64 years (Fig. 4A), for both sexes (Figs. 4B and 4C).

### Projected mortality trends

The designed predictive model projects 29,606 deaths for OCC (supplementary Table 1, Additional file 1) and 13,500 deaths for OPC in Spain for the period 2020–2044 (supplementary Table 2, Additional file 1). An increase in the number of deaths for OCC is expected from 5,408 (period 2020–2024) to 6,445 (period 2040–2044); instead, a decrease from 2,794 (period 2020–2024) to 2,596 (period 2040–2044) deaths for OPC is expected. Taking sex into account, 15,416 deaths in males and 14,191 in females are anticipated for OCC (male/female ratio: 1/1), and 10,667 deaths in males and 2,833 in females for OPC (male/female ratio: 4/1). The graphical representation of these data can be seen in Figs. 1 and 2.

Regarding the projected specific mortality rate per 100,000 inhabitants, the predictive model for OCC foresaw that it will drop from 2.07 (period 2020–2024) to 1.68 (period 2040–2044) (supplementary Table 3, Additional file 1), and for OPC it is expected to decrease from 1.11 (period 2020–2024) to 0.71 (period 2040–2044) (supplementary Table 4, Additional file 1). Considering sex, the predictive model estimated that the OCC mortality rate will decrease in males from 2.78 (period 2020–2024) to 2.10 (period 2040–2044). In females, a slight increase in mortality rate is expected, from 1.48 in the period 2020–2024 to 1.56 in the period 2040–2044. With respect to OPC, mortality rate in males will decrease from 2.02 (period 2020–2024) to 1.35 (period 2040–2044) according to the model. In females, a slight drop is estimated from 0.34 (period 2020–2024) to 0.28 (period 2040–2044). The graphical representation of these data depicts a levelling off and subsequent decline in OCC mortality rates (Fig. 3A). This decrease is more pronounced in males > 65 years (Fig. 3B), while a slight increase is expected in females, especially in > 65 years (Fig. 3C). Predicted OPC mortality rates show a levelling off and subsequent drop in all age groups through the end of the period (Fig. 4A). In males, the decrease in OPC mortality rates will be more evident in > 65 years (Fig. 4B), while in females there will be a constant increase in > 65 years until the period 2035–2039. This upward trend appears to be stabilizing at the end of the period (Fig. 4C).

### Comparison between OCC and OPC mortality trends

**Fig. 5:**
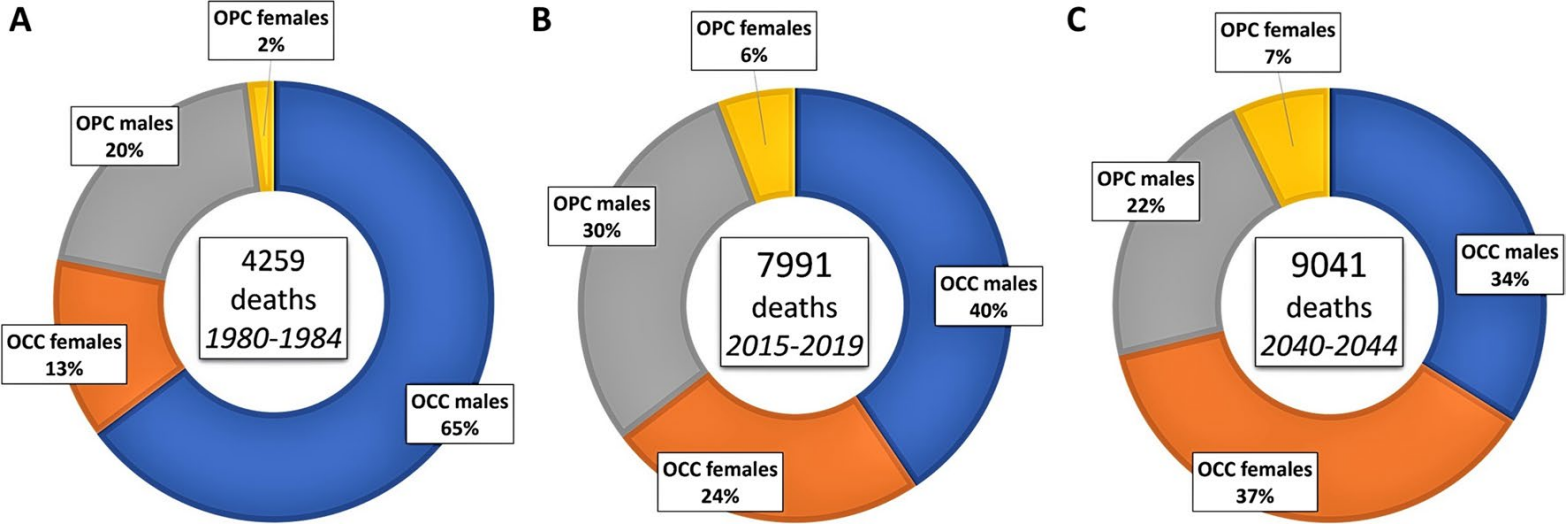
Proportion of total deaths for OCC and OPC, categorized by sex: **A** observed 1980–1984 period; **B** observed 2015–2019 period; **C** projected 2040–2044 period. The size of each donut is scaled to reflect the total number of deaths

**Fig. 6:**
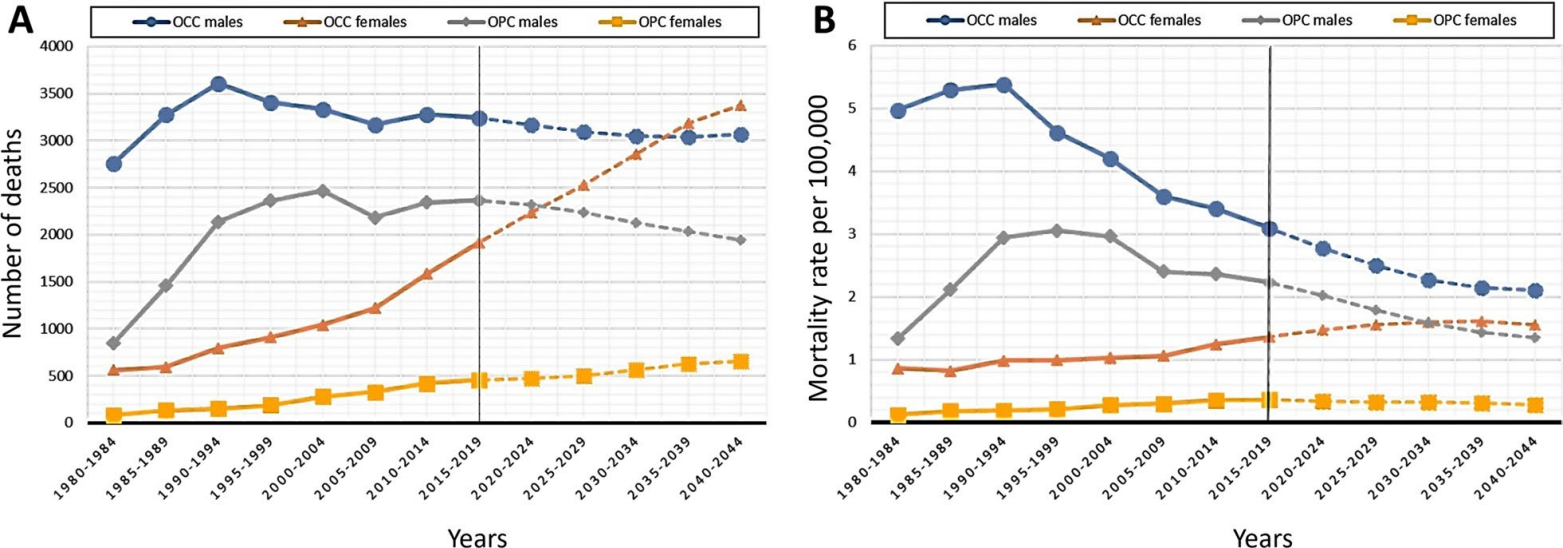
**A** Graphical representation of the number of observed (period 1980–2019) (solid line) and projected (period 2020–2044) (dash line) deaths for OCC and OPC, categorized by sex. **B** Graphical representation of observed (period 1980–2019) (solid line) and projected (period 2020–2044) (dash line) mortality rates per 100,000 inhabitants (European population 2013) for OCC and OPC, categorized by sex

The comparative balance of the number of deaths from OCC and OPC between the periods 1980–1984 and 2015–2019 showed a gross increase in mortality of 55% for OCC and 199% for OPC. Between the periods 2020–2024 and 2040–2044, the model estimated an upward trend in deaths for OCC (19%) and a downward trend for OPC (-7%). The death ratio between OCC/OPC exhibited a downward trend from 3.6 (period 1980–1984) to 1.8 (period 2015–2019) and 2.5 (period 2040–2044). Figure 5 shows the gross proportion of deaths for OCC and OPC in different study periods. In Fig. 6A, the estimated number of deaths for OCC and OPC are graphically compared by sex. The predictive model foresees that in the period 2040–2044 there will be a greater number of OCC deaths in females than in males. Furthermore, the number of deaths for OPC will decrease in males and gradually increase in females. The specific mortality rate for OCC will decrease in males and increase in females until the period 2035–2039 and then stabilize, while for OPC it will decrease in males and increase and stabilize in females (Fig. 6B).

## Discussion

This study reports, for the first time, a prediction of the temporal trend of OCC and OPC mortality for the next 25 years in Spain, analysed by sex and age groups. The most relevant findings according to our prediction model indicated that OCC mortality rates will tend to decrease in males while there will be a slight increase in females, more evident in those > 65 years. Regarding OPC mortality rates, the model predicts a downward trend in males, except in those > 65 years. In this group mortality rates will rose until the period 2025–2029, when they will begin to stabilize and then decrease. In contrast, OPC mortality in females will remain stable, showing a gradual increase in females > 65 years until the end of the period.

The possible association with the main known risk factors should be considered to explain the variations detected in mortality trend rates between OCC and OPC when analysing them according to sex and age groups. Traditionally, the main non-genetic risk factors for these related cancers are smoking and alcohol consumption, with synergistic effects when their use is combined [8]. The legislative and cultural changes of the late 1990s plausibly favoured a reduction in the population's exposure to tobacco and alcohol, which resulted in a decrease in mortality rates in those cancers associated with these etiological factors, in particular for OCC [9]. The global prevalence of OCC could be reduced by 75% by limiting tobacco and alcohol consumption in the population and increasing public awareness of its prevention [10]. In our study, these trend changes seemed to be clearly more noticeable in males, since a stabilization and decrease in OCC mortality is expected until the end of the study period. However, OCC mortality in females will increase throughout the analysed period, especially in females > 65 years, which is probably explained by their incorporation into tobacco consumption that occurred in Spain during the last decades of the twentieth century. The traditional gender difference in the prevalence of smoking has narrowed in Spain, due to an increase in the prevalence of female smokers, especially in the younger age groups [11]. Nevertheless, alcohol consumption in Spanish society remains constant in both sexes [11]. In gross figures, the number of OCC deaths in males in the period 2040–2044 is expected to reach similar values to those of the period 1980–1984; instead, the number of deaths in females is projected to increase by 490% between the periods 1980–1984 and 2040–2044. Our projections indicate that at the end of the estimated period, OCC mortality will reverse the classic trend of epidemic behaviour that associated this specific type of cancer with males, tobacco, and alcohol consumption, since it is estimated that by then, more females than males will die from OCC.

Regarding OPC mortality, our findings suggested that it will increase steadily in females over the next 25 years, although it will tend to stabilize. However, in females > 65 years, this increase is projected to continue until the end of the period studied. Since HPV infection is a well-established risk factor for a high proportion of oropharyngeal squamous cell carcinomas [12], it seems reasonable to assume that this increase in mortality could correspond to an increase in the incidence of HPV infection. Although information on HPV status was not available in our INE data, a causal association has been reported in the literature, as up to > 80% of OPC patients are infected with HPV [13]. The potential beneficial effect of HPV vaccination on OPC mortality remains unknown, but it is speculated that raising the awareness of the population and health professionals [14] together with institutional HPV vaccination campaigns will modify this specific risk factor. This could be especially important in younger age groups, in which mortality will tend to stabilize or decrease gradually. The long-term benefits of the HPV vaccine will largely depend on the duration of its protection [15]. Extensive vaccination campaigns with prolonged protection for at least 30 years are likely to be required to assess whether HPV vaccination has a significant impact on OPC mortality. The results of our study suggested that the impact of current HPV vaccination on OPC mortality trends in patients > 65 years will be quite limited over the next 25 years, and therefore the reduction in OPC mortality due to HPV vaccination among older adults will take longer [16].

The gross death rate comparison between OCC/OPC is projected to be halved from 6.2 (period 1980–1984) to 3.1 (period 2040–2044). When OCC and OPC mortality rates are compared by sex up to 2044, estimated projection predicts a strong decline in male mortality and an increase in females for OCC. This trend was especially relevant in females > 65 years, and according to the model it will reverse the classic mortality trends of OCC between males and females. Likewise, OPC mortality rates in males are expected to decline at the end of the period, while in females they will stabilize, except in those > 65 years where mortality rates are expected to rise. Therefore, our findings emphasized the need to maintain educational interventions aimed at the entire population in the coming years, promoting smoking cessation and alcohol abstinence, vaccination against HPV, campaigns for early diagnosis of potential malignant lesions in the oral cavity and oropharynx, changes in lifestyle, preservation of oral hygiene and periodontal health [17], and high consumption of fruits and vegetables. Our predictive model indicated that these preventive programs should be intensified especially in females in the near future.

Few studies on a global and European scale predicted mortality for the coming years. Kujan et al. [18] reported a projection analysis for OCC-OPC between 2012 and 2030 using data from GLOBOCAN 2012, estimating that the global mortality rate will increase by 50%. Thus, the burden projected by OCC-OPC between 2012 and 2030 will rise from 145,353 to 220,627 (for both sexes). In our model, the number of deaths from OCC-OPC in the period 2015–2019 was 7,991 deaths, and in the period 2040–2044 an increase of 8,912 deaths is projected, which represents an increase of 11.5% in Spain. Smittenaar et al. [19] estimated cancer cases and deaths between 2015 and 2035 in the UK. They found that while death rates for most cancers are declining, there will be an accelerating average annual increase in oral cancer death rates (1.42% for males, 1.53% for females), due to the most pronounced changes in the > 75 years group. A similar pattern is found for oral, anal, and cervical cancers, leading to speculation that similarities between these cancers are particularly associated with HPV. The number of deaths from oral cancer is expected to rise by 2.97% in males and 3.09% in females between 2014 and 2035. A study conducted in China has also shown an upward trend in future mortality rates from OCC-OPC [20].

## Limitations and strengths

This study presented some limitations that must be addressed. First, death certificates may be affected by errors that can influence subsequent analysis of mortality rates. However, it can be established that there have been no recent changes in classification and staging in subsequent revisions of the ICD. Mortality registries are more reliable than incidence registries, since the data they provide are usually more complete, better compiled and, therefore, more contextually real. For this reason, mortality certificates are a frequent and important source of public health studies and provide a tool to reliably evaluate public health interventions. Mortality data are the only ones that allow evaluating such a long population series, and in the case of cancer, previous studies have demonstrated the quality of these data [21]. Second, mortality predictions are necessarily affected by changes in cancer diagnosis or treatment, so they should be interpreted with caution if new treatment modalities may emerge in the coming years. Nevertheless, mortality trends are unlikely to be influenced by changes in diagnosis and certification in the near future, as these cancers are relatively easy to detect. Third, the standardization of rates has been carried out using the European standard population of 2013, so our data are sufficiently reliable and comparable to allow us to infer mortality trends in most of the European population, at least in South/Mediterranean countries. Nonetheless, the information on death certificates is not homogeneous or consistent between countries. Consequently, to improve our ability to predict mortality trends in the future, it would be desirable for death certificates to be uniform in all countries.

## Conclusions

This predictive model study provided a global view of OCC and OPC mortality trends up to 2044 in Spain. Our results have predicted future changes in mortality trends by sex, age, and topographic locations. Based on our findings, OCC mortality rates will tend to stabilize and decrease in males but will increase markedly in females > 65 years. However, OPC mortality rates will increase until the middle of the period in males and subsequently decrease, while in females > 65 years there will be an increase and stabilization until the end of the study period. The similarities and differences revealed by the mortality projections when comparing OCC and OPC can be explained by the variable influence on exposure to main known risk factors and the efficacy of the available therapeutic tools. Some of the risk factors involved can be prevented by campaigning for smoking cessation and alcohol abstinence. To the same extent that OCC mortality rates in males have declined in the last observed decades, there is still room to improve OCC mortality rates in females > 65 years in the future by strengthening campaigns against the risk factors mentioned above. OPC mortality in both sexes is going to become a growing health problem in the coming years. Vaccination campaigns for the prevention of HPV-associated malignant neoplasms could have a preventive effect on mortality trends that will have to be evaluated in future studies. Nevertheless, although the protective role of the HPV vaccine in the long-term prevention of these cancers is still unknown, the increase in mortality for OPC makes it advisable to conduct educational campaigns to inform the general population about HPV forms of transmission and its important etiopathogenic role in disease development. Our findings highlighted the importance of closely monitoring the mortality rates for both types of cancers by age group and sex, and the need to continue preventive measures against known risk factors.

## Supplementary Information

**Additional file 1**. The datasets that were used and analysed in the study.

## Data Availability

Mortality data for the period 1980–2019 were obtained from death certificates in the public domain free access at the Spanish National Institute of Statistics (http://www.ine.es). Population data for the period 2020–2044 were calculated on the basis of the official population projection figures provided in the public domain free access at the Spanish National Institute of Statistics (http://www.ine.es). The datasets used and/or analysed during the current study are provided in Additional file 1. Additional data and materials are available from the corresponding author on reasonable request.
