# Supplementary material for: Estimated projection of oral cavity and oropharyngeal cancer deaths in Spain to 2044
Source: BMC Oral Health. 2022 Oct 14;22:444. doi: 10.1186/s12903-022-02487-6 (PMC9563172; doi:10.1186/s12903-022-02487-6)
Supplement: Supplementary file 1 — Additional file 1. The datasets that were used and analysed in the study. [file 12903_2022_2487_MOESM1_ESM.pdf]

Additional File Material for:

Estimated projection of oral cavity and oropharyngeal cancer deaths in Spain to 2044

Pedro Infante-Cossio [1], Antonio-Jose Duran-Romero [2], Antonio Castaño-Seiquer [3], Rafael Martinez-De-Fuentes [4] and Jose-Juan Pereyra-Rodriguez [2]

[1] Department of Oral and Maxillofacial Surgery, Virgen del Rocio University Hospital, University of Seville, Seville, Spain
[2] Department of Dermatology, Virgen del Rocio University Hospital, University of Seville, Seville, Spain
[3] Department of Preventive and Community Dentistry, School of Dentistry, University of Seville, Seville, Spain
[4] Department of Prosthodontics, School of Dentistry, University of Seville, Seville, Spain

Content:

Table 1. Number of observed (period 1980-2019) and projected (period 2020-2044) deaths by 5-year blocks for OCC: A) both sexes; B) males; C) females.

Table 2. Number of observed (period 1980-2019) and projected (period 2020-2044) deaths by 5-year blocks for OPC: A) both sexes; B) males; C) females.

Table 3. Observed (period 1980-2019) and projected (period 2020-2044) age-specific mortality rates per 100,000 inhabitants (European population 2013) by 5-year blocks for OCC: A) both sexes; B) males; C) females. SMR: standardized mortality rates

Table 4. Observed (period 1980-2019) and projected (period 2020-2044) age-specific mortality rates per 100,000 inhabitants (European population 2013) by 5-year blocks for OPC: A) both sexes; B) males; C) females. SMR: standardized mortality rates

Table 1. Number of observed (period 1980-2019) and projected (period 2020-2044) deaths by 5-year blocks for OCC: A) both sexes; B) males; C) females.

NUMBER OF DEATHS BY ORAL CAVITY CANCER

	Observed deaths								Projected deaths				
Age (years)	1980-1984	1985-1989	1990-1994	1995-1999	2000-2004	2005-2009	2010-2014	2015-2019	2020-2024	2025-2029	2030-2034	2035-2039	2040-2044
**A) Both sexes**													
<34	70	40	23	32	30	27	31	40	56	57	63	69	71
35-44	178	213	226	218	215	134	86	81	111	135	183	184	197
45-54	673	643	720	703	661	641	553	438	341	373	406	473	581
55-64	850	1184	1218	1061	970	1010	1085	1136	1035	826	687	742	778
65-74	766	885	1142	1200	1191	1054	1131	1209	1362	1436	1381	1124	958
>75	787	903	1079	1103	1309	1523	1977	2259	2503	2796	3181	3638	3860
All ages	3324	3868	4408	4317	4376	4389	4863	5163	5408	5623	5901	6229	6445
**B) Males**													
<34	51	31	15	20	19	14	18	24	31	30	31	33	33
35-44	158	193	204	184	174	106	60	57	79	96	129	127	135
45-54	609	598	651	635	582	544	445	329	252	273	306	364	444
55-64	745	1078	1097	955	848	843	872	889	740	564	461	508	555
65-74	650	740	946	984	948	846	856	881	916	908	805	627	530
>75	546	633	697	628	762	815	1027	1062	1151	1226	1313	1383	1368
All ages	2759	3273	3610	3406	3333	3168	3278	3242	3168	3096	3045	3043	3064
**C) Females**													
<34	19	9	8	12	11	13	13	16	25	28	32	36	38
35-44	20	20	22	34	41	28	26	24	33	39	54	57	63
45-54	64	45	69	68	79	97	108	109	90	100	100	109	137
55-64	105	106	121	106	122	167	213	247	295	262	226	234	223
65-74	116	145	196	216	243	208	275	328	446	529	576	497	428
>75	241	270	382	475	547	708	950	1197	1352	1570	1867	2254	2493
All ages	565	595	798	911	1043	1221	1585	1921	2240	2528	2856	3186	3381

Table 2. Number of observed (period 1980-2019) and projected (period 2020-2044) deaths by 5-year blocks for OPC: A) both sexes; B) males; C) females.

NUMBER OF DEATHS BY OROPHARYNGEAL CANCER

	Observed deaths								Projected deaths				
Age (years)	1980-1984	1985-1989	1990-1994	1995-1999	2000-2004	2005-2009	2010-2014	2015-2019	2020-2024	2025-2029	2030-2034	2035-2039	2040-2044
**A) Both sexes**													
<34	11	14	10	10	5	2	6	5	6	5	6	6	6
35-44	70	126	168	208	153	84	60	29	45	64	59	59	62
45-54	244	396	536	599	649	549	480	350	232	195	237	287	269
55-64	303	548	802	807	816	807	895	926	772	586	431	386	461
65-74	200	331	488	607	741	634	705	864	959	947	833	649	486
>75	107	176	288	317	380	439	617	654	780	943	1134	1281	1312
All ages	935	1591	2292	2548	2744	2515	2763	2828	2794	2742	2700	2668	2596
**B) Males**													
<34	7	9	9	10	3	1	2	5	5	5	5	5	5
35-44	65	121	159	187	130	64	47	24	38	64	59	59	62
45-54	233	389	519	558	590	484	404	300	210	171	217	286	269
55-64	289	526	769	773	768	726	785	787	653	507	384	327	420
65-74	183	299	454	575	678	574	617	748	808	775	658	516	397
>75	76	115	228	257	296	335	488	504	607	716	808	844	789
All ages	853	1459	2138	2360	2465	2184	2343	2368	2321	2237	2130	2036	1943
**C) Females**													
<34	4	5	1	0	2	1	4	0	1	1	1	1	1
35-44	5	5	9	21	23	20	13	5	7	0	0	0	0
45-54	11	7	17	41	59	65	76	50	22	25	20	1	1
55-64	14	22	33	34	48	81	110	139	119	79	47	59	41
65-74	17	32	34	32	63	60	88	116	151	172	175	133	89
>75	31	61	60	60	84	104	129	150	173	228	326	438	523
All ages	82	132	154	188	279	331	420	460	473	504	570	632	654

Table 3. Observed (period 1980-2019) and projected (period 2020-2044) age-specific mortality rates per 100,000 inhabitants (European population 2013) by 5-year blocks for OCC: A) both sexes; B) males; C) females. SMR: standardized mortality rates

AGE-SPECIFIC MORTALITY RATES FOR ORAL CAVITY CANCER

	**Observed standardized mortality rate**								**Projected standardized mortality rate**				
Age (years)	1980-1984	1985-1989	1990-1994	1995-1999	2000-2004	2005-2009	2010-2014	2015-2019	2020-2024	2025-2029	2030-2034	2035-2039	2040-2044
**A) Both sexes**													
<34	0.11	0.12	0.06	0.07	0.05	0.05	0.07	0.12	0.18	0.19	0.20	0.21	0.21
35-44	1.00	1.07	1.15	0.97	0.86	0.48	0.26	0.24	0.37	0.48	0.78	0.78	0.79
45-54	3.03	3.19	3.51	3.04	2.68	2.34	1.83	1.47	0.98	1.04	1.28	1.64	2.22
55-64	4.58	5.65	5.67	5.28	4.66	4.25	4.18	4.00	3.31	2.50	1.85	1.97	2.30
65-74	5.73	6.18	6.92	6.45	6.10	5.57	5.77	5.47	5.57	5.18	4.31	3.22	2.44
>75	9.88	9.55	10.05	8.72	8.77	8.70	9.92	10.96	11.22	11.01	10.71	10.51	9.94
SMR	2.63	2.78	2.93	2.64	2.47	2.23	2.23	2.17	2.07	1.95	1.82	1.73	1.68
**B) Males**													
<34	0.17	0.18	0.07	0.08	0.07	0.07	0.10	0.13	0.21	0.20	0.19	0.20	0.20
35-44	1.85	1.97	2.11	1.68	1.39	0.76	0.36	0.33	0.51	0.67	1.08	1.06	1.04
45-54	5.57	6.08	6.41	5.58	4.75	4.03	2.97	2.21	1.41	1.44	1.84	2.46	3.30
55-64	8.50	10.72	10.65	9.85	8.41	7.29	6.90	6.46	4.88	3.51	2.53	2.76	3.36
65-74	11.35	11.92	12.84	11.64	10.62	9.67	9.30	8.53	8.10	7.24	5.73	4.17	3.15
>75	18.29	17.85	17.72	13.29	13.39	11.82	12.85	12.48	12.48	11.94	11.30	10.62	9.80
SMR	4.97	5.29	5.38	4.61	4.2	3.6	3.4	3.09	2.78	2.5	2.27	2.15	2.1
**C) Females**													
<34	0.07	0.06	0.05	0.06	0.05	0.05	0.07	0.12	0.17	0.19	0.22	0.23	0.24
35-44	0.19	0.18	0.21	0.27	0.33	0.19	0.16	0.14	0.21	0.27	0.42	0.44	0.45
45-54	0.59	0.42	0.71	0.57	0.64	0.68	0.70	0.74	0.50	0.54	0.60	0.71	0.97
55-64	1.11	1.01	1.09	1.10	1.16	1.37	1.60	1.66	1.85	1.60	1.22	1.24	1.30
65-74	1.52	1.88	2.27	2.22	2.35	2.10	2.69	2.78	3.59	3.81	3.80	3.19	2.43
>75	5.59	5.35	6.06	6.49	6.41	7.14	8.40	10.35	10.91	11.10	11.90	13.18	13.29
SMR	0.86	0.82	0.98	0.99	1.03	1.06	1.24	1.36	1.48	1.56	1.6	1.61	1.56

Table 4. Observed (period 1980-2019) and projected (period 2020-2044) age-specific mortality rates per 100,000 inhabitants (European population 2013) by 5-year blocks for OPC: A) both sexes; B) males; C) females. SMR: standardized mortality rates

AGE-SPECIFIC MORTALITY RATES FOR OROPHARYNGEAL CANCER

	Observed standardized mortality rate								Projected standardized mortality rate				
Age (years)	1980-1984	1985-1989	1990-1994	1995-1999	2000-2004	2005-2009	2010-2014	2015-2019	2020-2024	2025-2029	2030-2034	2035-2039	2040-2044
**A) Both sexes**													
<34	0.02	0.03	0.04	0.06	0.02	0.01	0.02	0.03	0.02	0.02	0.02	0.02	0.02
35-44	0.43	0.59	0.84	1.06	0.68	0.36	0.20	0.10	0.15	0.28	0.28	0.28	0.27
45-54	1.11	1.93	2.62	2.56	2.62	2.11	1.64	1.19	0.72	0.57	0.71	1.00	1.00
55-64	1.62	2.60	3.73	3.98	3.90	3.36	3.42	3.26	2.45	1.76	1.18	1.03	1.40
65-74	1.50	2.31	2.97	3.29	3.80	3.35	3.56	3.91	3.92	3.43	2.61	1.87	1.28
>75	1.29	1.79	2.57	2.45	2.51	2.40	2.97	3.03	3.28	3.50	3.58	3.37	2.95
SMR	0.67	1.07	1.46	1.53	1.53	1.28	1.29	1.23	1.11	0.99	0.87	0.78	0.71
**B) Males**													
<34	0.03	0.05	0.10	0.12	0.02	0.01	0.02	0.04	0.04	0.04	0.04	0.04	0.04
35-44	0.82	1.14	1.63	1.94	1.18	0.51	0.32	0.16	0.23	0.55	0.55	0.54	0.54
45-54	2.15	3.84	5.20	4.80	4.84	3.77	2.76	1.95	1.29	0.96	1.30	2.04	2.03
55-64	3.28	5.20	7.43	7.93	7.59	6.21	6.16	5.70	4.23	3.06	2.16	1.78	2.76
65-74	3.23	4.83	6.16	6.85	7.63	6.58	6.64	7.19	7.14	6.15	4.59	3.32	2.38
>75	2.56	3.21	5.68	5.47	5.28	4.82	5.99	5.95	6.33	6.66	6.68	6.11	5.15
SMR	1.33	2.11	2.94	3.05	2.96	2.4	2.36	2.23	2.02	1.79	1.58	1.44	1.35
**C) Females**													
<34	0.01	0.02	0.01	0.00	0.01	0.01	0.03	0.00	0.01	0.01	0.01	0.01	0.01
35-44	0.05	0.04	0.07	0.19	0.18	0.20	0.08	0.03	0.08	0.00	0.00	0.00	0.00
45-54	0.12	0.08	0.19	0.36	0.46	0.47	0.53	0.48	0.13	0.14	0.21	0.01	0.01
55-64	0.14	0.20	0.30	0.36	0.46	0.67	0.82	0.94	0.75	0.56	0.25	0.32	0.41
65-74	0.27	0.53	0.38	0.34	0.67	0.59	0.84	0.99	1.19	1.25	1.16	0.99	0.50
>75	0.71	1.24	0.92	0.88	0.97	1.03	1.04	1.16	1.23	1.45	1.91	2.36	2.65
SMR	0.12	0.18	0.19	0.21	0.28	0.3	0.35	0.36	0.34	0.33	0.33	0.31	0.28
